# Efficacy of Amblyopia Treatments in Children Up to Seven Years Old: A Systematic Review

**DOI:** 10.7759/cureus.56705

**Published:** 2024-03-22

**Authors:** Artashes Yeritsyan, Ashka V Surve, Bolaji Ayinde, Priyank Chokshi, Sanjeev Adhikari, Aniket Jaimalani, Pousette Hamid

**Affiliations:** 1 Medicine, California Institute of Behavioral Neurosciences & Psychology, Fairfield, USA; 2 Internal Medicine, California Institute of Behavioral Neurosciences & Psychology, Fairfield, USA; 3 Internal Medicine, Pandit Deendayal Upadhyay Medical College, Rajkot, IND; 4 Internal Medicine, Surat Municipal Institute of Medical Education and Research, Surat, IND; 5 Neurology, California Institute of Behavioral Neurosciences & Psychology, Fairfield, USA

**Keywords:** amblyopia treatment, ‏strabismus, dichoptic training, virtual reality, pharmacologic therapy, strabismus surgery, spectacle correction, occlusion therapy, patching, amblyopia

## Abstract

Amblyopia is a neurodevelopmental disorder of the visual system that impairs the vision of millions of children worldwide. Amblyopia is best treated within the sensitive period of visual development when a child is up to seven years of age. Currently, the gold standard for early treatment of childhood amblyopia is patching, with new treatments emerging in recent years. We aim to evaluate the effectiveness of these newly developed treatments for amblyopia in children aged seven years and younger while comparing them to the current industry standard of patching. We searched online databases including PubMed, Google Scholar, and Cochrane Library for randomized controlled trials (RCTs), systematic reviews, meta-analyses, and narrative reviews relating to amblyopia treatment in children aged seven and younger. We only included articles and studies completed within the last five years and those written in the English language. After compiling a list of 297 articles, we removed duplicates, articles without an available full text, and those not relevant to our topic. Of the remaining 51 articles, we were left with 22 after reading abstracts and removing further irrelevant articles. We did a quality assessment on the remaining 22 articles and were left with 14 articles for our systematic review after removing eight low-quality articles. Of the 14 articles, we had eight RCTs, two systematic reviews, one comparative interventional study, and three narrative reviews. Seven of the articles contained data reinforcing the effectiveness of patching while comparing it to other treatment modalities. Three of the articles had data supporting spectacle correction, including a novel form called alternative flicker glass which delivers occlusion therapy via a spectacle frame with unique lenses, and ultimately deemed it at least as effective or more than patching. Data from three articles supported the use of surgery to successfully correct the angle of strabismus. Findings from five articles backed the use of pharmacologic therapy, specifically atropine when used alongside patching as a more effective alternative to patching solely. However, levodopa plus patching had no advantage over patching alone. Additionally, seven articles addressed the use of virtual reality (VR) and dichoptic therapy as prospective treatments for childhood amblyopia. VR therapy proved beneficial when used within one week after strabismus surgery. Dichoptic training was also effective in improving amblyopic-eye visual acuity when used on its own or in conjunction with spectacles. Furthermore, dichoptic movie therapy was found to be more effective than patching. Thus, we found multiple highly effective treatments for childhood amblyopia that are as effective or more than patching. Future studies should consider prescribing these treatments to larger cohorts while also performing a cost-benefit analysis for each treatment. In addition, more needs to be learned about the potential adverse side effects of these treatments, especially for pharmaceutical therapy.

## Introduction and background

Every year 2-3% of children are born with amblyopia, a developmental disorder of the visual system that impairs vision in one and rarely both eyes [1,2]. Amblyopia is thought to develop during the early stages of a child's life, specifically during the critical period of visual development [3]. There are multiple forms of amblyopia including strabismus, form deprivation, and refractive [4]. Strabismus is most often referred to as “lazy eye” as one or rarely both eyes deviate outwards/laterally (exotropia) or inwards/medially (esotropia). Form deprivation amblyopia occurs when an obstruction prevents or blocks light from entering and reaching the retina of the eye where all the photoreceptors are located. This is the rarest form of amblyopia and is most often seen via congenital cataracts but can also be due to corneal scarring or ptosis. Refractive amblyopia occurs when there is a refractive error between the eyes causing a difference in focus [4]. A significant unilateral refractive error is considered anisometropic amblyopia, meanwhile, a significant bilateral refractive error is considered isometropic amblyopia [5].

Previous studies have shown that the treatment of amblyopia in children is best accomplished before the age of seven years, as this is a critical period of visual development [6-8]. As such, the first seven years in a child’s life are a crucial period for the detection and proper treatment of amblyopia. For decades the gold standard for the treatment of childhood amblyopia has been standard occlusion therapy, more commonly known as patching [9,10]. Until recently, advancements have been made in the early detection and treatment of amblyopia, specifically using virtual reality (VR) movie and game therapy, surgery, pharmacologic therapy, and advanced spectacles. However, since there is a narrow treatment period, not much is known about the efficacy of these novel treatments for amblyopia in children aged seven years and younger, as most amblyopic children within this age group are treated with either standard occlusion therapy or spectacle correction as first-line treatment [10,11]. Although previous studies and systematic reviews have individually addressed the treatments outlined above, we aim to have a comprehensive review of the efficacy of the most novel forms of treatment for childhood amblyopia during the sensitive period of visual development, while comparing it to the traditional treatment of standard occlusion therapy.

## Review

Methodology

Our systematic review was conducted using Preferred Reporting Items for Systematic Reviews and Meta-Analysis (PRISMA) guidelines [12].

Database and search strategy

We started our search on June 25, 2023, and our database consisted of online libraries. For our data collection, we searched PubMed, Google Scholar, and Cochrane Library. We searched for studies that focused on the treatment of amblyopia for children/preschoolers. Our strategy included using keywords and medical subject headings (MeSH) such as amblyopia, strabismus, lazy eye, esotropia, exotropia, treatment, therapy, children, and preschool. The outcomes from these searches are recorded in Table 1.

**Table 1:** Search results This table displays search results found for keywords and MeSH (Medical Subject Headings) keywords from PubMed, Google Scholar, and Cochrane Library.

Keyword/MeSH keyword	Database	Number of search results
Amblyopia	PubMed	10,372
	Google Scholar	125,000
	Cochrane Library	14
Lazy eye	PubMed	10,384
	Google Scholar	268,000
	Cochrane Library	8
Strabismus	PubMed	26,315
	Google Scholar	366,000
	Cochrane Library	15
Exotropia	PubMed	3,355
	Google Scholar	51,300
	Cochrane Library	5
Esotropia	PubMed	4,574
	Google Scholar	63,400
	Cochrane Library	3
Anisometropic	PubMed	1,232
	Google Scholar	12,800
	Cochrane Library	0
Amblyopia, treatment	PubMed	5,603
	Google Scholar	66,300
	Cochrane Library	14
Strabismus, treatment	PubMed	14,896
	Google Scholar	172,000
	Cochrane Library	13
Amblyopia, children	PubMed	6,235
	Google Scholar	62,700
	Cochrane Library	13
Strabismus, children	PubMed	13,542
	Google Scholar	184,000
	Cochrane Library	11
Amblyopia, treatment, children	PubMed	3,874
	Google Scholar	46,800
	Cochrane Library	13
Amblyopia, treatment, preschool	PubMed	2,440
	Google Scholar	8,870
	Cochrane Library	11
Strabismus, treatment, children, preschool	PubMed	4,927
	Google Scholar	13,900
	Cochrane Library	6
Amblyopia, treatment, therapy, children, preschool	PubMed	2,186
	Google Scholar	9,290
	Cochrane Library	8
Amblyopia, strabismus, lazy eye, exotropia, esotropia, treatment, therapy, children, preschool	PubMed	80
	Google Scholar	286
	Cochrane Library	0

Inclusion criteria

We reviewed articles and studies only from the following classifications: randomized controlled trials (RCTs), systematic reviews, meta-analyses, and traditional reviews (narrative reviews). We only selected peer-reviewed articles written in the English language, and articles and studies done within the previous five years.

Exclusion criteria

We excluded case reports/series, case-control studies, gray literature, observational studies, editorials, and animal studies. We also excluded studies that did not include full-text articles and studies done more than five years ago.

Quality assessment

We used the Cochrane risk-of-bias tool for randomized trials (RoB 2) to assess the quality of RCTs. Additionally, we used the Scale for the Assessment of Narrative Review Articles (SANRA) questionnaire for narrative/traditional reviews, and A MeaSurement Tool to Assess systematic Reviews (AMSTAR) to assess the quality of systematic reviews. After performing a quality assessment, we omitted low-quality studies.

Data collection

After gathering a final list of articles upon quality assessment, data was independently collected by the first author. All other authors assisted in the analysis and interpretation of the data.

Results

**Figure 1:**
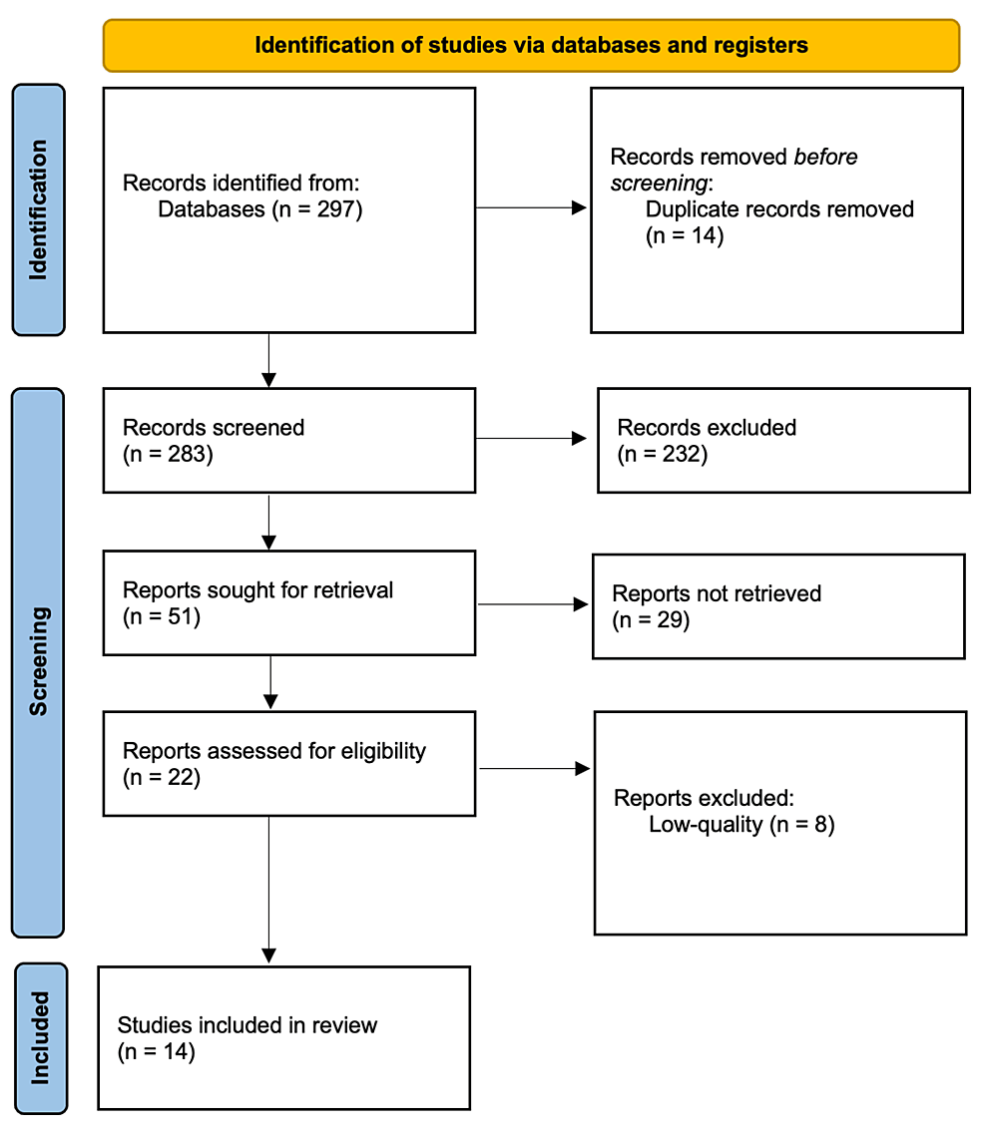
PRISMA flow diagram PRISMA: Preferred Reporting Items for Systematic Reviews and Meta-Analysis

We compiled a list of the 297 most relevant studies based on relevant keywords in the title (i.e., amblyopia, treatment, and/or child). Of the 297 studies, 14 were duplicates leaving us with 283 studies. We then took a closer look at the titles and excluded articles that were not relevant to our study (e.g., did not include children, included children above seven years old, or discussed the prevalence of amblyopia and not treatment). This left us with 51 relevant studies. We then looked deeper into these studies, reading the abstracts. Of the 51 abstracts, 12 did not include full-text articles. Now leaving us with 39 articles, we excluded 17 articles after reading them as we found them not relevant to our study specifically. Left with 22 articles, we then conducted a quality assessment. We excluded 8 low-quality articles and included 14 articles for data extraction (Figure 1). A summarized table of study characteristics of the 14 articles is included in Table 2.

**Table 2:** Characteristics of studies used for data extraction RCT: Randomized controlled trial; VR: Virtual reality; AFG: Alternative flicker glass; CAPT: Combined atropine and patching therapy; BNT: Botulinum neurotoxin; I-BiT: Interactive binocular treatment; VA: Visual acuity; BCVA: Best corrected visual acuity; CSF: Contrast sensitivity function; y/o: Years old; LogMAR: Logarithm of the Minimum Angle of Resolution

Author	Journal and publication year	Title	Study/article type	Type of amblyopia	Treatment(s)	Conclusion	Total # of patients	# of males	# of females
Zhang et al. [13]	Indian J Ophthalmol. 2023	The effect of virtual reality technology in children after surgery for concomitant strabismus	RCT	Strabismus (exotropia & esotropia)	VR	Improved binocular vision function (near stereovision acuity), post-surgical eye position maintenance, and cure rate	200	97	103
Yuan et al. [14]	Ophthalmic Res. 2021	Alternative Flicker Glass: A New Anti-Suppression Approach to the Treatment of Anisometropic Amblyopia	RCT	Anisometropic	AFG vs. patching alone	Improved BCVA, CSF, and stereoacuity in spectacle-wearing group	40	22	18
Xiao et al. [15]	Ophthalmology 2022	Randomized Controlled Trial of a Dichoptic Digital Therapeutic for Amblyopia	RCT	Strabismus, Anisometropic, or Mixed	Dichoptic therapy	Improved BCVA	105	60	45
Wang et al. [16]	JAMA Ophthalmol. 2021	Effect of Combined Atropine and Patching vs Patching Alone for Treatment of Severe Amblyopia in Children Aged 3 to 12 Years: A Randomized Clinical Trial	RCT	Strabismus, Anisometropic, or Mixed	CAPT vs. patching alone	CAPT group had more mean amblyopic-eye VA improvement	108	54	54
Song et al. [17]	BMC Ophthalmol. 2022	Comparison of alternate part-time patching and pencil push-up training for patients with intermittent exotropia	RCT	Strabismus (exotropia only)	patching vs. pencil push-ups	Both groups significantly improved distance control but no significance between the groups	92	45	47
Park [18]	Korean J Ophthalmol. 2019	Current Management of Childhood Amblyopia	Narrative Review	Strabismus, Anisometropic, or Mixed	spectacle correction, patching, pharmacologic penalization (atropine), occlusion glasses, perceptual learning, dichoptic training	All improved VA on varying degrees	NA	NA	NA
Mayet et al. [19]	Eye (Lond). 2021	Botulinum neurotoxin injections in essential infantile esotropia-a comparative study with surgery in large-angle deviations	RCT	Strabismus (esotropia only)	BNT vs. surgery	Improved esotropia angle	101	40	61
Manny et al. [20]	Optom Vis Sci. 2022	A Randomized Trial of Binocular Dig Rush Game Treatment for Amblyopia in Children Aged 4 to 6 Years	RCT	Strabismus, Anisometropic, or Mixed	dichoptic binocular game with spectacle correction vs spectacle correction alone	Greater improvement in amblyopic-eye VA in dichoptic binocular game group at four-week mark	182	86	96
Li et al. [21]	Cochrane Database Syst Rev. 2019	Conventional occlusion versus pharmacologic penalization for amblyopia	Systematic Review	Strabismus, Anisometropic, or Mixed	atropine penalization vs. conventional occlusion (patching)	Both as effective at improving amblyopic-eye VA	1177	NA	NA
Jost et al. [22]	Sci Rep. 2022	Randomized clinical trial of streaming dichoptic movies versus patching for treatment of amblyopia in children aged 3 to 7 years	RCT	Strabismus, Anisometropic, or Mixed	dichoptic movies vs. patching	Improved amblyopic-eye BCVA in both groups. Dichoptic movie group had additional BCVA improvements up to six weeks	60	26	34
Farvardin et al. [23]	J Ophthalmic Vis Res. 2019	Levodopa Plus Occlusion Therapy versus Occlusion Therapy Alone for Children with Anisometropic Amblyopia	Comparative Interventional Study	Anisometropic	levodopa plus occlusion therapy vs. occlusion therapy alone	Both groups had similar improvements in logMAR. Levodopa administration with occlusion therapy gave no additional logMAR improvements	40	NA	NA
Dong et al. [24]	Int J Ophthalmol. 2021	Surgery at early versus late for intermittent exotropia: a Meta-analysis and systematic review	Systematic Review	Strabismus (exotropia only)	early and late strabismus surgery	Patients below four y/o experienced better long-term outcomes from early strabismus surgery	NA	NA	NA
Chaturvedi et al. [25]	Indian J Ophthalmol. 2023	Binocular vision therapy for the treatment of Amblyopia—A review	Narrative Review	Strabismus, Anisometropic, or Mixed	Dichoptic iPad/iPod-based training, VR (I-BiT)	Playing dichoptic iPad/iPod-based video games previously showed improvement in amblyopic-eye VA. However, this study found no statistically significant improvement as the patients had received previous occlusion therapy.	NA	NA	NA
Azizalrahman [26]	J Med, L & Public Health. 2022	Video Games in the Treatment of Amblyopia: A Narrative Review	Narrative Review	Strabismus, Anisometropic, or Mixed	Dichoptic video games and movies	Dichoptic video games provided the greatest visual function improvement in children under seven years old	NA	NA	NA

Study characteristics

For our data analysis, our data encompassed eight RCTs, two systematic reviews, one comparative interventional study, and three narrative reviews.

Discussion

Amblyopia Treatment

Since amblyopia is a developmental disorder of the visual system, it interrupts proper stimuli perception from one, or seldom both, eyes. In doing so, the brain experiences difficulty perceiving the stimuli from the amblyopic eye and thus “shuts off” the eye and relies on obtaining easy-to-read stimuli from the non-amblyopic dominant eye, also known as the fellow eye [27]. Patients with amblyopia often experience poor visual acuity (VA), contrast sensitivity, and decline or loss of stereopsis [28-30]. Thus, the aim of amblyopia treatment is to correct or improve these deficiencies to improve the quality of life of amblyopic patients.

Addressing ocular dominance and providing each eye with a clear image on the retina are the fundamentals of treating amblyopia. Treatment possibilities include traditional occlusion therapy via patching the fellow eye, spectacle correction, pharmacologic penalization, and various novel VR therapy options. After the diagnosis of amblyopia in children, it is crucial to begin early treatment as a significant improvement of visual function is best achieved in children under seven years of age [6,18].

The Gold Standard

The gold standard for the treatment of amblyopia is standard occlusion therapy or patching. Patching involves placing an eye patch over the fellow eye in order to promote the use and activation of the amblyopic eye. In doing so, it forces the brain to work in alignment with the amblyopic eye to perceive a clear image on the retina. In short, it forces the child to use their amblyopic eye to perceive clear images. Depending on the severity of the child’s amblyopia, doctors currently recommend two to six hours of daily patching to improve amblyopic-eye VA in children [31,32].

Of our chosen 14 articles, five directly tested the use of patching against other treatment modalities for strabismus, anisometropic, or mixed conditions. Of those studies, all reconfirmed patching as an effective treatment for childhood amblyopia [14,16-18,22].

In an RCT by Yuan et al., 20 children with anisometropic amblyopia were instructed to wear a traditional patch on their fellow eye for two hours a day. In 12 weeks, their mean best corrected visual acuity (BCVA) improved by 0.18 ± 0.18 logMAR (95% CI = 0.09-0.26). Additionally, their contrast sensitivity function (CSF) improved significantly. It should be noted that their mean Titmus stereoacuity did seem to improve but was not significant (p > 0.05) [14].

In another RCT by Wang et al., 55 children with strabismus, anisometropia, or mixed amblyopia were instructed to wear an eye patch on their fellow eye for six hours a day. In addition to wearing the eye patch, these participants were instructed to complete activities at near-visual distances, such as painting, for a minimum of one hour daily. After six months, the mean amblyopic eye VA improved by 0.58 logMAR which is approximately 5.8 lines on the Snellen chart. Additionally, while none of the children had stereoacuity at baseline, 12 children (approximately 22%) achieved a stereoacuity of 400 arcsec or better at six months [16]. Similar results were found where children with severe amblyopia, having a VA of 20/100 to 20/400, achieved a VA improvement of 3.6 lines after 17 weeks of daily two-hour patching. Furthermore, increasing the daily patching time to six hours was found to improve VA after a period of unvarying VA [18]. Likewise, in a recent study from 2022, Jost et al. found that two weeks of two hours of daily patching resulted in significant amblyopic-eye VA improvement by 0.06 ± 0.05 logMAR (p < 0.0001) [22].

More recently, in 2022, Song et al. conducted an RCT investigating the efficacy of patching versus pencil push-ups for the treatment of exotropia in children three to seven years old. Although this study did not measure VA/BCVA, stereoacuity was measured and no significant improvement was found in either group. However, after 12 weeks, distance control significantly improved in both the patching and pencil push-up groups (from 2.8 ± 1.1 points to 1.6 ± 1.0 points and from 3.1 ± 1.1 to 2.0 ± 1.5 points, respectively) [17]. Both of these home-based treatments, patching and pencil push-ups, are often highly favored by ophthalmologists and optometrists as early forms of treatment of amblyopia [18,33]. Given the limited effective treatment period for childhood amblyopia, and since there was no significant improvement in stereoacuity, it seems that it would be much more beneficial for children to undergo alternative therapies.

In short, although all five studies reconfirmed the effectiveness of patching for the treatment of amblyopia in children up to seven years old, most also found better alternatives.

Correction of Refractive Error

Along with patching, spectacle/optical correction is one of the most widely used methods of first-line treatment of amblyopia [11,34]. Spectacles work by aligning the eyes or by bringing objects into clear view and thus balancing VA between the eyes [35]. In a 2022 study, to test the efficacy of spectacle correction versus a dichoptic binocular game for the treatment of amblyopia in children under seven years old, 90 children aged four to six years old were prescribed to wear spectacles for eight weeks. By four and eight weeks, mean amblyopic-eye VA improved by 0.6 logMAR lines and 1.0 logMAR lines, respectively. While mean amblyopic-eye VA improved significantly, the same cannot be said for stereoacuity as the median change of stereoacuity was 0 arcsec at both four and eight weeks [20]. Similar results of improved binocular BCVA via spectacle correction were found in numerous studies as addressed by Park [18].

Similarly, in 2021 a novel form of anti-suppression treatment for anisometropic amblyopia was tested on 20 children. Alternative flicker glass (AFG) is a unique treatment modality as it administers occlusion therapy via a spectacle frame with lenses made of liquid crystal glass. These unique lenses consist of two thin plates that are overlaid with a polarized film. In between the two plates lies a gel with organic molecules inside. Occlusion is achieved by an electronic shutter that is activated by a microchip. At three weeks, the mean amblyopic-eye BCVA was 0.37 ± 0.20 logMAR (95% CI = 0.27-0.46) which was a significant improvement (p < 0.01) when compared to baseline mean BCVA of 0.45 ± 0.20 logMAR (95% CI = 0.35-0.54). Incredibly, by 12 weeks mean BCVA further significantly improved (p < 0.01) to 0.28 ± 0.19 logMAR (95% CI = 0.19-0.37). It should be noted that this same study also prescribed 20 children to patching therapy to test the efficacy of AFG in comparison to traditional occlusion therapy as referenced above. Yuan et al. found that mean BCVA was not significantly different between the AFG and patching groups at baseline, three-week, and 12-week follow-ups. However, between the two groups, the AFG group experienced larger improvements in stereoacuity and CSF of 6, 12, and 18 cycles per degree spatial frequencies [14]. Although this study should further be replicated with a larger participant population, an argument can be made that AFG is more effective than traditional patching at improving visual function.

Surgery

For patients suffering specifically from strabismic amblyopia, surgery has long been the gold standard for treatment. The aim of strabismus surgery is to adjust the extrinsic ocular muscles by tightening or loosening them, in order to realign the “wandering” eye to its correct position [36]. For children between six months and six years of age with a baseline angle of esotropia ≤60 PD, Mayet et al. found that 17 of 24 (70.8%) subjects who underwent bilateral medial rectus muscle recession surgery achieved orthophoria or a misalignment angle of less than 10 PD [19]. The efficacy of strabismus surgery also depends on when the surgery is performed. A 2021 meta-analysis found that patients below the age of four, with intermittent exotropia, experienced a better long-term outcome from early strabismus surgery [24]. Even though surgery has proved effective time and time again for strabismic children, roughly 6.7% are forced to undergo reoperation within one year of surgery, after experiencing regression of esotropia/exotropia angle [37]. Recent advancements have increased the success of this treatment method. One of these advancements is the use of VR technology in children after strabismus surgery. In a recent RCT, 200 children with a mean age of slightly over six years old were divided into two groups. Both groups received strabismus surgery, but the experimental group (N = 100) received VR training within one week of the procedure, while the control group (N = 100) did not. Children who used VR technology for six months experienced improved binocular vision function, near stereovision acuity, and cure rate. They also maintained post-surgical eye position better than the non-VR-using group [13]. Thus, even though strabismus surgery is an effective tool in the treatment of childhood strabismus, the efficacy of this tool is greatly enhanced when used in conjunction with post-surgical VR training technology. Future studies can be done to test this technology on children after undergoing congenital cataract extraction surgery.

Pharmacologic Therapy

Another method of occlusion therapy is the use of pharmacologic therapy such as eye drops, injections, or pills to occlude the fellow eye. A novel form of this therapy includes Botulinum neurotoxin (BNT) injections. In an RCT for strabismic children between six months and six years of age, Mayet et al. found that 13 out of 26 (50%) children, with baseline angle of esotropia ≤60 PD, who received up to three, 5 unit, injections of BNT achieved orthophoria or a misalignment angle of less than 10 PD. It should be noted that of the original BNT cohort of 54 children (26 with baseline angle of esotropia ≤60 PD and 28 with baseline angle of esotropia >60 PD), 27 received no response from BNT and had to undergo strabismus surgery to improve their ocular misalignment angle. Although BNT was not as useful for some children, it was deemed effective since it takes five times less time than surgery to reach similar results, making it a possible treatment method in countries with fewer ophthalmic surgical resources [19]. However, further research with larger populations is needed to investigate adverse side effects and to optimize this treatment before it can be considered a viable alternative to strabismus surgery.

Another pharmacologic therapy, levodopa, a precursor to dopamine, aims to improve visual function by reducing the size of the retina’s receptive field. To experimentalize this hypothesis, researchers gathered a cohort of 40 children aged six to seven years old with hyperopic anisometropic amblyopia. Two groups of 20 children each, received three hours of daily occlusion therapy with the experimental group receiving levodopa and the control group receiving a placebo pill. Treatment was administered for three weeks. Twelve weeks after treatment termination both groups had similar improvements in logMAR. Thus, levodopa administration with occlusion therapy gave no additional improvements in logMAR and visual outcome benefits [23].

Atropine drops have long been used in ophthalmology and optometry clinical offices before certain eye examinations. Atropine can also be used for fellow eye occlusion for the treatment of childhood amblyopia. A systematic review from 2019, which took into account seven trials and a total of 1177 amblyopic eyes, found that patching alone and atropine penalization alone were both as effective at improving amblyopic-eye VA [21]. More quantitatively, another study found that for children under seven years of age, atropine penalization and patching alone improved VA by 3.16 lines and 2.84 lines respectively [18]. Furthermore, in a 2021 RCT, 53 amblyopic children were prescribed eye patching along with one drop of atropine sulfate (1%) once daily for the first three days, then once every two days for the remainder of the experiment. Concurrently, 55 amblyopic children were prescribed patching alone. After six months, the combined atropine and patching therapy (CAPT) group had more mean amblyopic-eye VA improvement than the patching-only group, validating the efficacy of patching alongside the use of atropine drops against patching alone [16]. Due to the efficacy and low-cost nature of these drops, it seems that they have high potential to be incorporated alongside patching as a first-line treatment for childhood amblyopia.

Virtual Reality/Dichoptic Therapy

The uses of VR therapy and dichoptic therapy as novel forms of treatment for childhood amblyopia have gained much popularity in recent years. The ability to have dichoptic training, that is, presenting simultaneous yet separate stimuli to each eye, is what makes VR so intriguing as it bypasses the forced occlusion of the fellow eye seen with traditional occlusion therapy [38,39]. As stated before, the use of VR therapy has already been shown to maintain post-surgical eye position, improve binocular vision function, near stereovision acuity, and cure rate when used within one week of strabismus surgery [13]. In a similar fashion, the use of dichoptic therapy has been shown to be effective when used in conjunction with spectacle correction. In a 2022 RCT, 92 amblyopic children aged four to six years old were prescribed to play a one-hour daily dichoptic iPad game, Dig Rush, for five days a week while also wearing prescribed spectacles during all waking hours. Compared to the control group of 90 similarly aged children who were prescribed spectacle correction only and achieved mean amblyopic-eye VA improvement of 0.6 logMAR lines, the dichoptic therapy group experienced a greater improvement of 1.1 logMAR lines after four weeks [20]. Similar results were found in children four to seven years old, where 12 weeks of one hour daily, six days a week, home-based dichoptic digital therapeutics plus full-time spectacle use, improved amblyopic-eye VA by 1.8 lines. This is a statistically significant (p = 0.0011) improvement compared to the control group who only wore full-time spectacles and saw an improvement by 0.8 lines [15].

Dichoptic therapy has also been shown to be beneficial for amblyopia treatment on its own. In a 2022 RCT, 30 children aged three to seven years old were instructed to watch dichoptic animated movies on a Nintendo 3DS XL for approximately eight hours every two weeks. Simultaneously, a control group of similar size and age was prescribed for patching therapy only. By two weeks, both groups had similar amblyopic-eye BCVA improvement, showing that dichoptic movie therapy is at least as beneficial as patching. However, by four and six weeks, the movie group had additional BCVA improvements of 0.13 ± 0.11 logMAR and 0.15 ± 0.10 logMAR, respectively. Additionally, after the initial two weeks, the patching group was switched to dichtopic movies and saw similar further BCVA improvements to the original movie group [22]. Thus, although both groups initially experienced similar improvements in amblyopic-eye BCVA, dichoptic movie therapy ultimately proved more effective as it showed additional BCVA improvements up to six weeks [22]. Additionally, recent studies have shown that amblyopic children experience similarly significant improvements in VA from both dichoptic video games and movies, with another study finding that after playing a binocular iPad game, children under seven years old experienced better and longer lasting improvement in VA when compared to children 7-13 years of age [26]. However, contrary to the previous studies [22,26], a Pediatric Eye Disease Investigator Group (PEDIG) trial of 385 amblyopic children found that when exposed to either one hour/day of dichoptic iPad games or two hours/day of patching therapy, the patching group achieved 0.31 more lines of mean amblyopic-eye VA improvement [18].

Interactive binocular treatment (I-BiT) is another emerging treatment modality for childhood amblyopia, as children are exposed to dichoptic stimuli via playing games or watching movies in VR. Even before the availability of shutter glasses, and spectacles that alter dimness in concurrence with the monitor, early studies revealed VA improvements in children who used I-BiT systems. Subsequently, after the availability of shutter glasses, a study found no statistical difference in amblyopic-eye VA improvement between children exposed to 30 minutes/week of dichoptic video games, movies/videos, or non-dichoptic video games. However, it should be noted that 76% of the 75 children, aged four to eight years old, enrolled in this study had received previous patching therapy and thus had impacted prospects of VA improvement [25].

Limitations

Despite having 14 articles as data resources, our systematic review does impose some limitations. We only included RCTs, systematic reviews, meta-analyses, and narrative reviews. We excluded studies published prior to the last five years, and those that did not include full-text articles nor were written in the English language. We further excluded gray literature, case reports/series, editorials, and animal studies. Additionally, because the studies involved young cohorts, some studies expressed limitations and dropouts due to poor adherence to protocol by the children. Before we can encompass some of the non-traditional forms of treatment into standard practice, further studies with larger population pools should take into account adherence to treatment and strategies to enhance treatment compliance. Additionally, more research should be conducted testing the efficacy of these treatments on visual function improvements other than VA (e.g., stereopsis and contrast sensitivity). Further studies should also take into consideration the cost-benefit analysis of each treatment type as cost can vary considerably.

## Conclusions

We examined the effectiveness of novel childhood amblyopia treatments for children up to seven years old in comparison to traditional occlusion therapy via patching. Although our results reaffirmed the efficacy of patching, we also discovered various alternatives as effective or more. Spectacle correction was found to be at least as effective or more than patching, with AFG showing additional benefits in CSF and stereoacuity. Furthermore, surgery and BNT injections both were highly beneficial as they significantly reduced the angle of exotropia/esotropia. Unlike the use of CAPT which was found to provide more VA improvements than patching alone, the use of levodopa with patching was not found to provide additional benefits in VA and visual function when compared to standard occlusion therapy. Additionally, the use of atropine drops alone were similarly effective in improving amblyopic-eye VA when compared to patching alone. Last, the uses of VR and dichoptic training were found to increase amblyopic-eye VA and showed promising results as potential treatments on their own or in conjunction with other treatment modalities. The use of VR after strabismus surgery was found to maintain post-surgical eye position and improve binocular vision function in addition to other vision benefits. Dichoptic movie and game training was found to be more effective than patching at improving amblyopic-eye BCVA. Although these studies reveal compelling outcomes, future studies with larger population pools must be conducted to expose possible adverse side effects, particularly for pharmacologic therapy before we can incorporate these nontraditional treatments into everyday medical practice.
